# Surgical outcomes of 215 patients with thymic epithelial tumors: A single‐center experience

**DOI:** 10.1111/1759-7714.13464

**Published:** 2020-05-08

**Authors:** Wenxin Tian, Yaoguang Sun, Qingjun Wu, Peng Jiao, Chao Ma, Hanbo Yu, Chuan Huang, Hongfeng Tong

**Affiliations:** ^1^ Department of Thoracic Surgery, Beijing Hospital, National Center of Gerontology Institute of Geriatric Medicine, Chinese Academy of Medical Sciences Beijing China

**Keywords:** Prognosis, thymectomy, thymic epithelial tumor

## Abstract

**Objectives:**

To evaluate the oncological prognosis and neurological outcomes for patients with thymic epithelial tumors (TETs) after thymectomy.

**Methods:**

Consecutive patients with TETs who underwent thymectomy at Beijing Hospital from January 2011 to December 2018 were retrospectively enrolled into the study. Clinical, pathological, and perioperative data was collected. Patients were followed‐up by telephone interview and outpatient records. Statistical analyses were performed using SPSS version 19.0.

**Results:**

A total of 215 patients (115 men and 100 women) were included in this study of which 133 patients (61.9%) had TETs associated with myasthenia gravis (MG), and 82 patients (38.1%) had thymic tumors without MG. A total of 194 (90.2%) patients were successfully followed‐up. The median follow‐up period was 42 months. The five‐year overall survival (OS) rate was 88.6%. MG was the first cause of death for patients with MG (6/10). Prognosis in MG patients was similar to those without MG. Multivariate Cox regression analysis demonstrated that TNM stage III + IV was an independent risk factor for OS. Incomplete resection and younger age were risk factors for tumor recurrence. For patients with MG, the cumulative complete stable remission (CSR) rate increased with the postoperative follow‐up period, and the five‐year CSR rate was 44.7%. Univariate Cox analysis indicated that age, preoperative MG duration and preoperative medication might correlate with CSR. Multivariate Cox analysis only indicated older age as a negative factor of achieving CSR.

**Conclusions:**

MG had little influence on OS and tumor recurrence of thymic tumors. The new TNM staging system was an independent prognostic factor. Incomplete resection and younger age were risk factors for tumor recurrence. Older age was a negative factor of achieving CSR for thymoma patients with MG after extended thymectomy.

**Key points:**

**Significant findings of the study**

MG was not a prognostic factor for thymic tumors. The new TNM staging system was useful for prediction of prognosis. Incomplete resection and younger age were risk factors for tumor recurrence. Older age led to a lower probability of achieving CSR for thymomatous MG.

**What this study adds**

This study had a relatively large sample size of patients with thymic epithelial tumors in a single center. We evaluated not only the oncological prognosis, but also neurological outcomes after thymectomy, which was a more comprehensive assessment of surgical effect for thymic epithelial tumors.

## Introduction

Thymic epithelial tumors (TET), including thymomas and thymic carcinomas, are derived from thymic epithelial cells. TET is the most common primary tumor of the anterior mediastinum, but is a relatively uncommon tumor. The incidence of thymic tumor is estimated to be at 3.9 per 1 000 000 in China,^1^ while in the Surveillance, Epidemiology, and End Results (SEER) database of 2015, the incidence has been reported as 2.6–2.9 per 1 000 000.^2^ Thymomas are always associated with myasthenia gravis (MG), which is one kind of autoimmune diseases with a neuromuscular junction (NMJ) disorder characterized by fluctuating weakness of skeletal muscles. Few single centers have a large scale of patients with thymic tumors. To evaluate the oncological prognosis and neurological outcomes, we reviewed 215 patients with thymic tumors at the Department of Thoracic surgery of Beijing Hospital from January 2011 to December 2018.

## Methods

### Patient selection

Consecutive patients with thymic epithelial tumors (TETs) who received surgical treatment at Beijing Hospital from January 2011 to December 2018 were retrospectively collected. A total of 215 patients (115 men, 100 women) were included. Clinical and pathological information including perioperative data, World Health Organization (WHO) classification, and TNM staging was collected. The diagnosis of MG was confirmed through clinical presentation, pharmacologic characteristics and electromyography. MG grading was according to Osserman classification system.

### Surgical techniques

Patients with MG received extended thymectomy, which was defined as the removal of the tumor and thymic gland with the mediastinal fat tissue bilaterally between the phrenic nerves, from the root of neck to the diaphragm. Patients without MG received thymectomy, which was defined as the removal of the tumor and the entire thymic gland. Adjacent lymph nodes were routinely removed simultaneously. Surgical procedures included unilateral video‐assisted thoracoscopic surgery (VATS) and transsternal thymectomy. For VATS thymectomy, the right lateral approach with three ports utilizing single lung ventilation was mainly adopted, and when the tumor was located on the left side of the mediastinum, left lateral VATS was adopted. Transsternal thymectomy was chosen when the tumor was larger than 5 cm or adjacent organs were invaded. Also, surgeon preference was another important factor for determining the surgical procedure. Surgery was defined as radical (R0) when a complete tumor resection was performed, and incomplete in case of microscopically (R1) or macroscopically (R2) residuals.

### Postoperative treatment

For patients with MG, preoperative medications were resumed about six hours after surgery. If oral administration was not permitted, nasal feeding was recommended. Patients with MG were advised to continue outpatient or hospitalization treatment for MG in the Neurology Department after discharge from the Thoracic Surgery Department.

Patients with thymic tumors of advanced stage or high‐grade malignancy according to postoperative pathology or incomplete resection (R1/R2 resection) were suggested for postoperative adjuvant radiotherapy. Patients with TNM stage IV or R2 resection were suggested for postoperative chemotherapy.

### Follow‐up

Follow‐up information including tumor recurrence and survival status, MG medications, and postintervention MG status was collected mainly by telephone interview and outpatient records. Patients underwent chest computed tomography (CT) scans every six months for the first two years, and annually thereafter or depending on clinical demand. Recurrence was divided into three categories including local (anterior mediastinum), regional (intrathoracic recurrence not contiguous with thymus or previous thymoma), and distal recurrence (intraparenchymal pulmonary nodules, extrathoracic recurrence), according to the International Thymic Malignancy Interest Group (ITMIG) criteria.^3^ The postoperative MG effect was evaluated according to the Myasthenia Gravis Foundation of America (MGFA) criteria defining the post intervention status.^4^

### Statistical analysis

Statistical analyses were performed using SPSS version 19.0 statistical software. Continuous data are presented as mean ± standard deviation or median and range. Categorical data are presented as number (percentage, %). The data of two groups were compared using a *t*‐test, χ^2^ test, or Fisher's exact test, as required. Overall survival and cumulative complete stable remission (CSR) rate was estimated using the Kaplan‐Meier method. The survival comparison of two groups was estimated using the log‐rank method. Uni‐ and multivariate Cox regression analysis were performed to analyze prognostic factors of survival, recurrence‐free status and CSR status. *P*‐values <0.05 were considered statistically significant.

## Results

### Patient characteristics

A total of 215 patients (115 men and 100 women) were included in this study. The mean age was 52.7 years (15–83 years). A total of 133 patients (61.9%) had thymic tumors associated with MG, of whom 26 patients were Osserman stage I, 33 stage IIa, 55 stage IIb, 12 stage III, and seven stage IV. To control MG, 101 patients (75.9%) were treated only with pyridostigmine bromide (90–480 mg/day) preoperatively, and 19 with pyridostigmine bromide and glucocorticoids or immunosuppressants. A total of 11 patients received preoperative immunoglobulin, 17 patients had tumors associated with other autoimmune diseases, and seven patients had a history of other tumors. Two patients was diagnosed with a second tumor at the same time.

### Perioperative and pathological data

A total of 127 (59.1%) patients received VATS thymectomy, and one was converted to thoracotomy due to the tumor invading the great vessels, while 88 (40.9%) patients received transsternal thymectomy. The resection was extended to the pericardium in 13 patients, lung in 18 patients, innominate vein in three patients, and the diaphragm in two patients. Simultaneously, one patient received lobectomy due to lung cancer, one received resection of thyroid cancer, and one received coronary artery bypass grafting (CABG). One patient died of ventilator‐associated pneumonia in the second month after VATS thymectomy. There were no other perioperative deaths. A total of 30 patients (14%) had perioperative complications, half of whom were considered to have postoperative myasthenia crisis (POMC). Patients experiencing POMC were all Osserman stage IIb–IV and WHO type B thymoma. The perioperative and pathological data are listed in Table 1.

**Table 1:** The perioperative and pathological data of thymic tumors

Variables	Data
n	215
Age (years old)	52.7 ± 13.7
Gender (Male/female)	115/100
With/without MG	133/82
Diameter (cm)	4.5 ± 2.2
Measured/predicted FEV1 (%)	81.1 ± 18.8
Surgical procedure (n [%])	
VATS	127 (59.1%)
Transsternal	88 (40.9%)
Operation time (minutes)	124.8 ± 47.9
Blood loss (mL)	133.6 ± 290.7
Postoperative drainage (days)	3.6 ± 1.7
Length of postoperative stay (days)	9.2 ± 7.5
TNM stage (n [%])	
I	157 (73.0%)
II	6 (2.8%)
IIIA	39 (18.1%)
IIIB	2 (0.9%)
IVA	10 (4.7%)
IVB	1 (0.5%)
WHO classification (n [%])	
A	10 (4.7%)
AB	41 (19.1%)
B1	43 (20.0%)
B2	64 (29.8%)
B3	12 (5.6%)
Mixed B types	26 (12.1%)
Thymic carcinoma	14 (6.5%)
MNT^†^	4 (1.9%)
U^‡^	1 (0.5%)
Resection status (n [%])	
R0	207 (96.3%)
R1	3 (1.4%)
R2	4 (1.9%)
Perioperative complications (n)	30 (14.0%)
Myasthenic crisis	15
Bleeding	2
Deep vein thrombosis	2
Cardiac events	6
Surgical site infection	2
Pneumonia	1
Chylothorax	1
Lower limb artery thromosis	1

† MNT, micronodular thymoma with lymphoid.

‡ U, unknown. The WHO type could not be distinguished.

Patients with thymic tumors with MG were younger than patients without MG (49.6 ± 13.7 vs. 57.6 ± 12.3 years). WHO type B2 (36.8%) and B1 (24.1%) were the most common types for patients with MG, while WHO type AB (28.0%) was the most common in patients without MG. No patients with thymic carcinoma or type A thymoma had MG. The two groups had a different distribution of WHO types (*P* = 0.000), but a similar distribution of TNM stage (*P* = 0.934). Comparison between patients with MG and without MG is listed in Table 2.

**Table 2:** Comparison between patients with myasthenia gravis (MG) and without MG

Variables	Patients with MG	Patients without MG	T or x^2^ value	*P*‐value
N	133	82		
Age (years old)	49.6 ± 13.7	57.6 ± 12.3	4.321	0.000
Gender (Male/female)	48/34	67/66	0.244	0.263
Other autoimmune disorders				
Diameter (cm)	4.4 ± 2.2	4.7 ± 2.3	0.855	0.394
Surgical procedure (n [%])				
VATS	78 (58.6%)	49 (59.8%)	0.872	0.887
Transsternal	55 (41.4%)	33 (40.2%)		
Operation time (minutes)	127.6 ± 41.9	120.3 ± 56.4	−1.077	0.283
Blood loss (mL)	134.7 ± 310.3	131.7 ± 257.5	−0.074	0.941
Postoperative drainage (days)	3.6 ± 1.6	3.5 ± 2.0	−0.299	0.765
Length of postoperative stay (days)	9.4 ± 8.0	8.8 ± 6.7	−0.582	0.561
TNM stage (n [%])				
I	97 (72.9%)	60 (73.2%)	0.147	0.702
II	5 (3.8%)	1 (1.2%)		
IIIA	23 (17.3%)	16 (19.5%)		
IIIB	1 (0.8%)	1 (1.2%)		
IVA	6 (4.5%)	4 (4.9%)		
IVB	1 (0.8%)	0 (0.0%)		
WHO classification (n [%])				
A	0 (0.0%)	10 (12.2%)	56.866	0.000
AB	18 (13.5%)	23 (28.0%)		
B1	32 (24.1%)	11 (13.4%)		
B2	49 (36.8%)	15 (18.3%)		
B3	9 (6.8%)	3 (3.7%)		
Mixed B types	24 (18.0%)	2 (2.4%)		
MNT	1 (0.8%)	3 (3.7%)		
Thymic carcinoma	0 (0.0%)	14 (17.1%)		
Resection status				
R0	129 (97.0%)	79(96.3%)	0.068	0.794
R1	2 (1.5%)	1 (1.2%)		
R2	2 (1.5%)	2 (2.4%)		
Complications (n [%])^†^	12(9.0%)	8 (9.6%)	0.023	0.879

† POMC were excluded for the comparison of perioperative complications between patients with and without MG.

### Oncological outcomes

**Figure 1:**
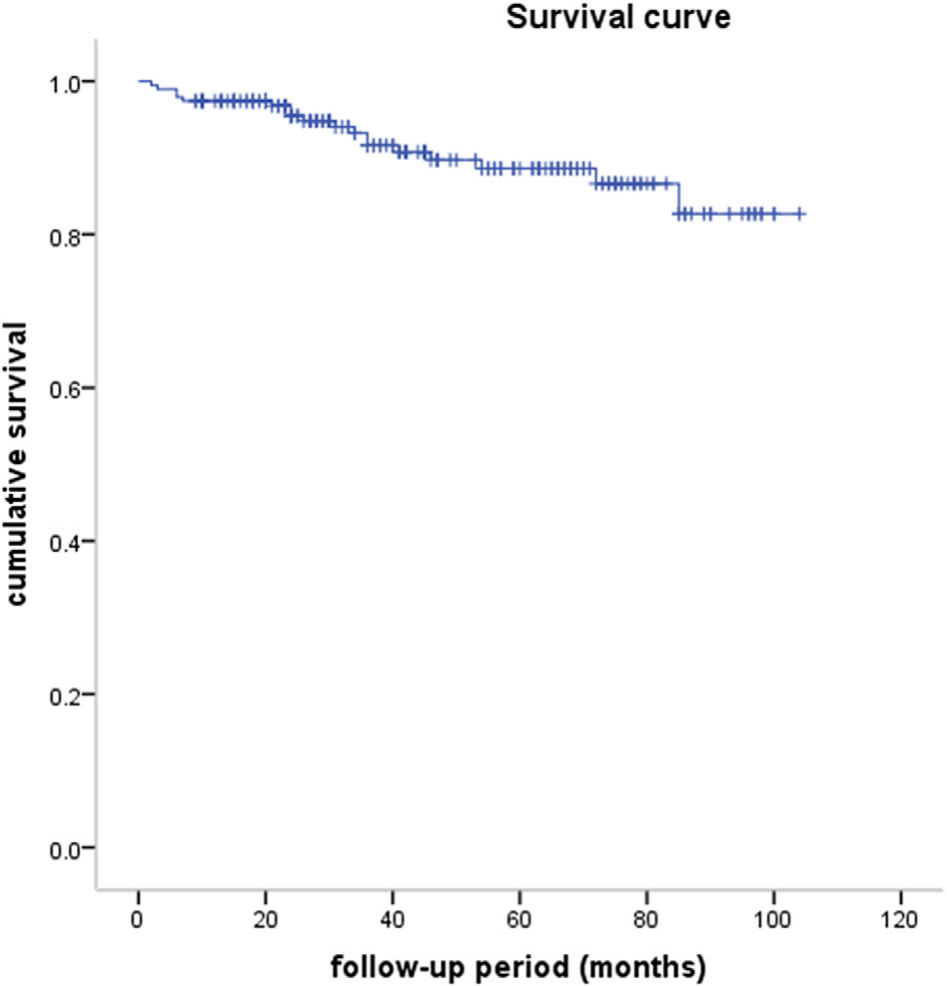
Survival curve of thymic epithelial tumors. (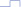) survival curve, (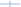) censored

**Figure 2:**
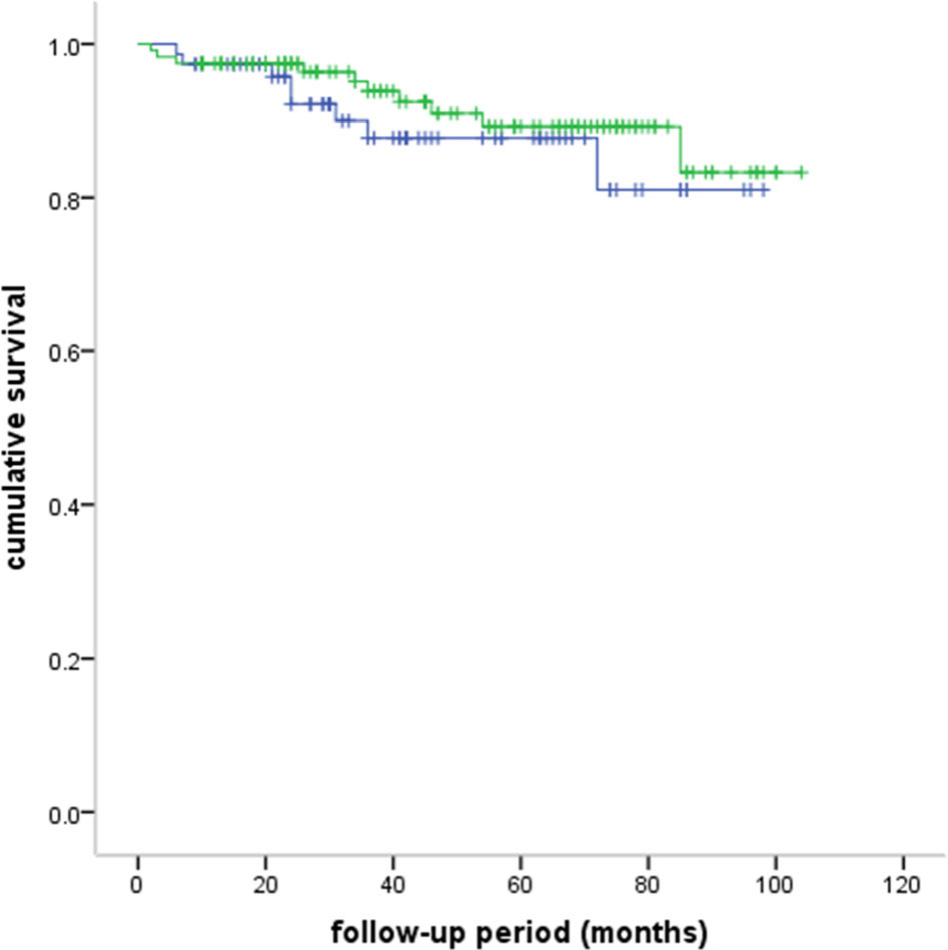
Comparison of survival curves between patients with MG and without MG (five‐year OS: 89.3% vs. 87.8%, *P* = 0.435). (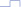) without MG, (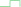) with MG, (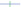) without MG censored, (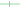) with MG censored

**Figure 3:**
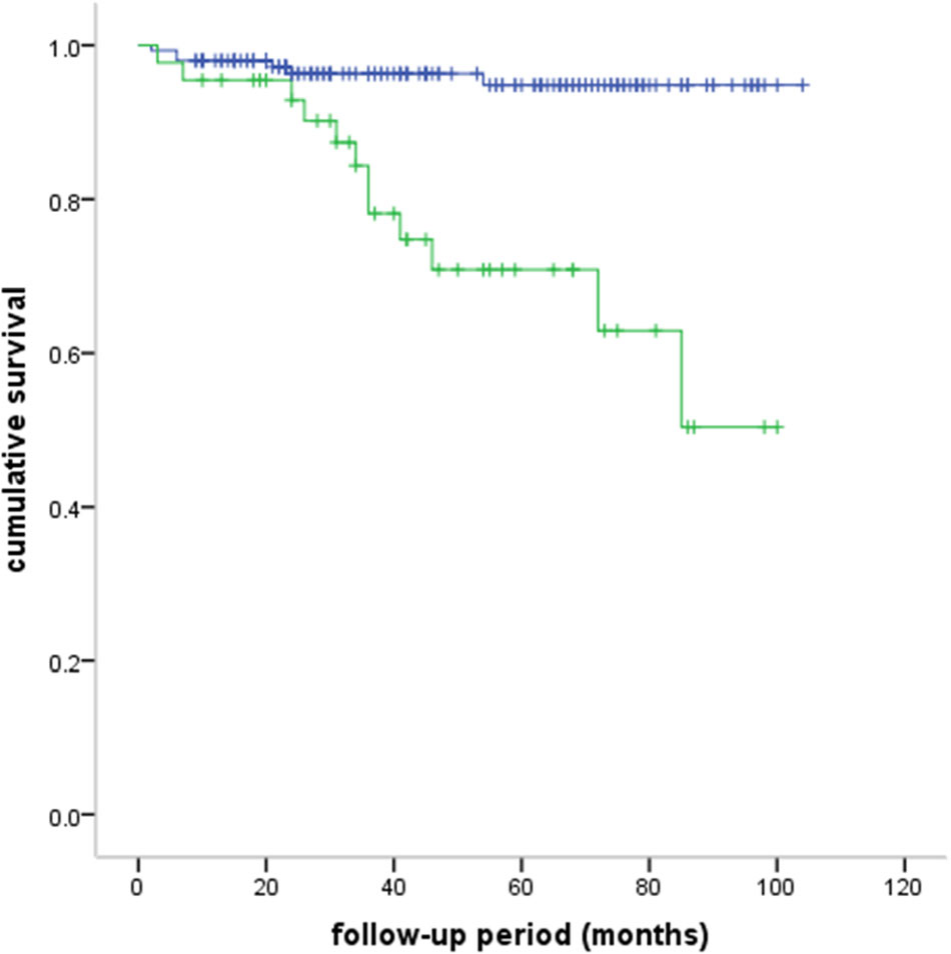
Comparison of survival curves between TNM stage I + II and stage III + IV (five‐year OS: 94.8% vs. 70.8%, *P* = 0.000). (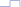) I + II, (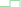) III + IV, (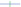) I + II‐censored, (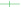) III + IV‐censored

**Figure 4:**
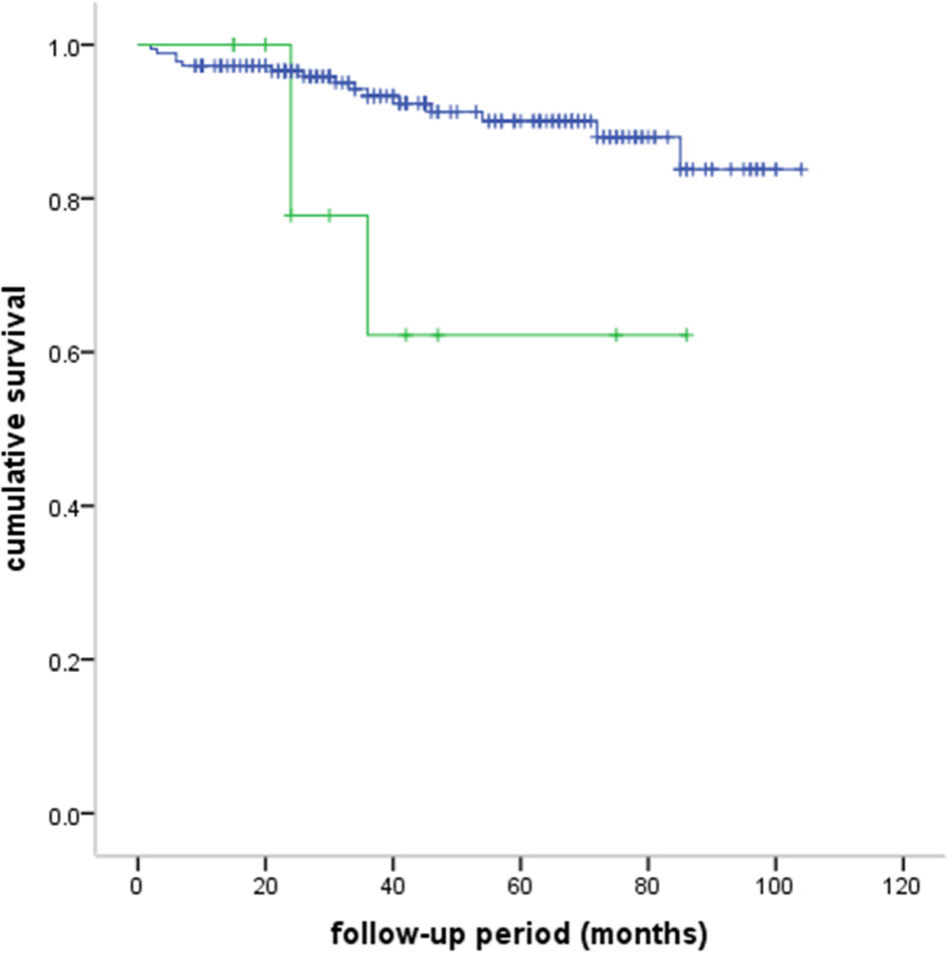
Comparisons of survival curves between thymoma and thymic carcinoma (five‐year OS: 90.1% vs. 62.2%, *P* = 0.022). (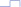) thymoma, (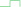) thymic carcinoma, (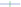) thymoma censored, (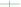) thymic‐carcinoma‐censored

A total of 112 patients received postoperative adjuvant therapy, of whom 104 received radiotherapy, four received chemotherapy, and four received radiotherapy and chemotherapy. A total of 194 (90.2%) were successfully followed‐up. The median follow‐up period was 42 months (range: 2–104 months). The five‐year OS rate was 88.6% (Fig 1). A total of 15 (7.7%) recurrences were observed; two patients had local recurrence, nine regional recurrence, and four distal recurrence. A total of 18 patients died: six of MG, seven of tumor recurrence, two of pneumonia, one of cardiac infarction, one of rectal cancer, and one unknown. The different groups of survival curves are shown in Figs 2, 3, 4.

For patients with thymomas, variables that were considered clinically relevant or showed a *P*‐value < 0.2 in univariate Cox regression analysis were entered into a multivariate Cox proportional‐hazards regression model. The Cox regression analysis demonstrated that TNM stage III + IV was an independent risk factor for OS (HR 6.944, 95% CI: 1.967–24.512, *P* = 0.003) (Table 3). Incomplete resection was a risk factor for tumor recurrence (HR 4.784, 95% CI: 1.067–21.450, *P* = 0.041), while older age was a protective factor (HR 0.930, 95% CI: 0.880–0.984, *P* = 0.012)(Table 4).

**Table 3:** Multivariate Cox regression analysis of risk factors for overall survival (OS)

Variable	HR	95% CI	*P*‐value
Resection status			0.276
R0	Reference	—	
R1/R2	2.149	0.542–8.522	
Surgical procedure			0.714
OT	Reference	—	
VATS	1.260	0.366–4.338	
TNM stage			0.003
I + II	Reference	—	
III + IV	6.944	1.967–24.512	
Complication			0.097
No	Reference	—	
Yes	2.469	0.849–7.179	

**Table 4:** Multivariate Cox regression analysis of risk factors for recurrence

Variable	HR	95% CI	*P*‐value
Age (continuous variable)	0.930	0.880–0.984	0.012
Resection status			0.041
R0	Reference	—	
R1/R2	4.784	1.067–21.450	
Complication			0.165
No	Reference	—	
Yes	2.533	0.682–9.404	
Postoperative therapy			0.088
No	Reference	—	
Yes	6.372	0.762–53.318	

### Neurological outcomes

**Figure 5:**
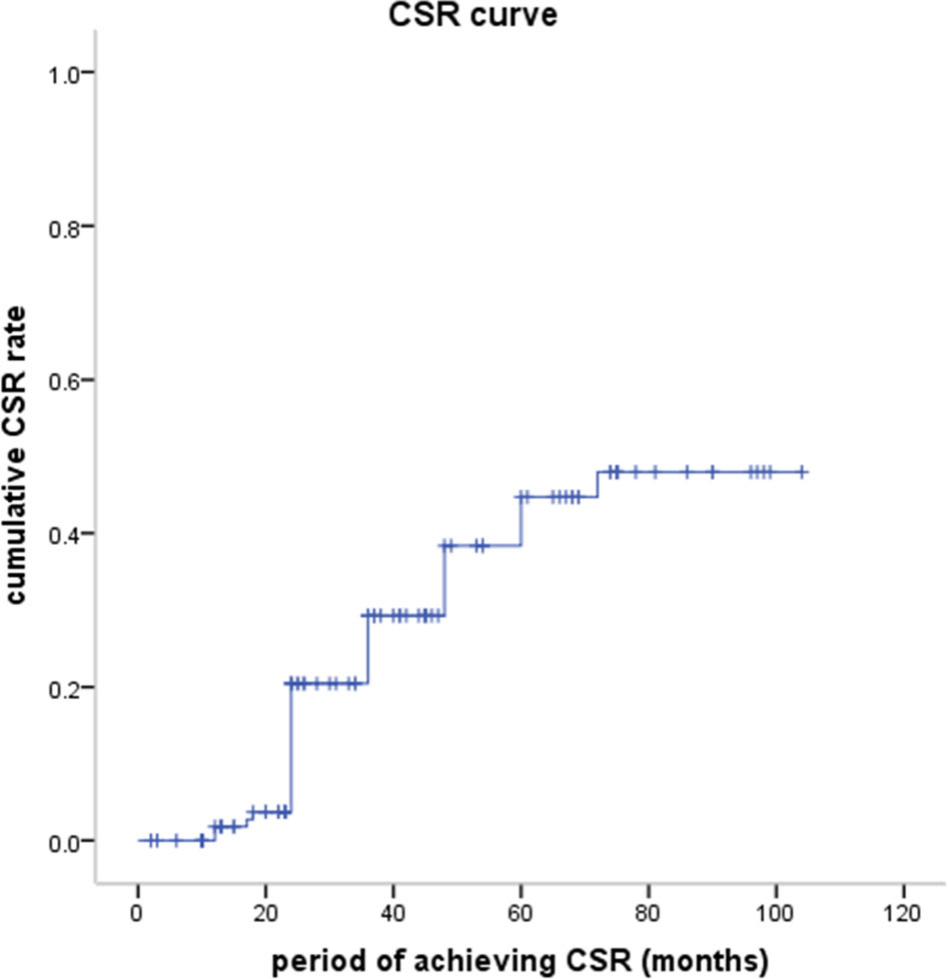
Cumulative CSR rate of patients with MG. (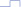) CSR curve, (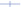) censored

I mean that in our study, patients who did not have MG before thymectomy, had not developed postoperative MG during follow‐up period. The postoperative status of MG was evaluated in a total of 116 patients (116/133, 87.2%). By the end of follow‐up, 35 patients (30.2%) had achieved complete stable remission (CSR), four patients (3.4%) achieved pharmacologic remission (PR), 33 patients (28.4%) had minimal manifestations (MM), 25 patients (21.6%) improved (I), six patients (5.2%) were unchanged (U), four patients (3.4%) were worse (W), in three patients (2.6%) there was exacerbation (E), and six patients (5.2%) died of MG (D). The cumulative CSR rate increased with the postoperative follow‐up period, and the five‐year CSR rate was 44.7% (Fig 5). The overall effective rate (CSR + PR+ MM + I) was 83.6% (97/116). CSR was chosen as the primary endpoint to analyze the predictive factors for CSR. Univariate Cox analysis demonstrated that age, preoperative MG duration and preoperative medications might correlate with CSR, while multivariate Cox analysis indicated that older age was a negative factor for achieving CSR (HR 0.976, 95% CI: 0.953–1.001, *P* = 0.055) (Table 5).

**Table 5:** Multivariate Cox regression analysis of risk factors for CSR

Variable	HR	95%CI	*P*
Age (continuous variable)	0.976	0.953–1.001	0.055
Symptom duration before operation			0.197
<6 months	Reference	—	
≥6 months	0.501	0.175–1.434	
Preoperative medications			0.188
None or pyridostigmine bromide only	Reference	—	
Steroid or immunosuppressants	0.449	0.136–1.481	

#### Discussion

Epidemiological data demonstrate that thymic epithelial tumors (TET) occur in approximately 10%–20% of patients with myasthenia gravis (MG) while, on the other hand, 25%–45% of patients with thymic tumors are associated with MG.^5^, ^6^ Moreover, 4%–7% of thymoma patients with MG have more than one paraneoplastic syndrome.^7^ In this study, MG occurred in 61.9% of patients with thymic tumors. Many patients diagnosed with MG are referred to our hospital because of our extensive experience in the treatment of MG patients, the high percentage of MG patients seen, and those subsequently diagnosed with thymic tumors after CT scanning.

The interaction between thymoma and MG is still controversial. Some experts^8^ have proposed that MG increases perioperative mortality, and MG is the main cause of death from thymomas with MG during the follow‐up period. However, MG symptoms may lead to early diagnosis of thymoma, which increases the rate of complete resection. Therefore, MG may be a protective factor for survival.^9^, ^10^ In this study, MG was the first cause of death for thymoma patients with MG (6/10). However, multivariate Cox regression analysis demonstrated that MG had little effect on survival or tumor recurrence. The controversial results of different studies may be due to different characteristics of patients admitted to each medical center and a small sample size.

The eighth edition of the TNM staging system for TET was proposed by the International Association for the Study of Lung Cancer (IASLC) and the International Thymic Malignancy Interest Group (ITMIG), and first published in 2014. The new TNM stage system not only describes the extent of tumor invasion, but also provides information on lymphatic involvement and tumor dissemination.^11^ Former studies have demonstrated that Masaoka‐Koga stage is a reliable predictive factor of OS and tumor recurrence.^9^, ^12^, ^13^ However, few studies have evaluated the clinical implementation of the TNM staging system. Ried *et al*.^14^ declared that the new TNM staging system presented a clinically useful and applicable system, which could be used for the prediction of prognosis for OS and recurrence‐free survival (RFS). This new staging system was used in our study, and multivariate Cox analysis indicated that TNM stage III + IV was the independent risk factor of OS, but did not significantly influence tumor recurrence. More prospective studies should be conducted to evaluate the value of stage‐adapted therapy and prediction of prognosis for thymic tumors.

Complete resection is quite an important factor in thymoma prognosis and tumor recurrence.^9^, ^13^ This study demonstrated that resection status did not significantly influence the survival, but incomplete resection increased the possibility of tumor recurrence. The role of WHO type is controversial for predicting overall survival.^12^, ^15^, ^16^ Thymic carcinomas are rare epithelial malignancies with a different biological behavior than thymomas. Patients with thymic carcinoma always have a poorer prognosis than those with thymoma, as observed in this study. However, our results did not indicate WHO types as a prognostic factor for OS of patients with thymomas. It has not been confirmed whether age is a predictive factor for OS and recurrence. Li *et al*.^17^has reported that older patients have a lower risk of recurrence, which is similar to our results. However, it should be pointed out that age was analyzed as a continuous variable in this study, while it may be switched to a categorical variable in other studies.

Neurological outcomes are quite important for the quality of life for thymoma patients with MG. Complete stable remission (CSR) means that the patient has had no symptoms or signs of MG for at least one year and has received no therapy for MG during that time. The CSR rate for MG patients with thymoma has been reported to be 16%–59.5% postoperatively, and the effective rate 75%–90%.^18^, ^19^, ^20^ MG patients with thymoma are quite different from those without thymoma in pathogenesis, clinical characteristics and treatment. MG patients with thymoma may have more severe symptoms, and poorer postoperative MG effect than those with thymic hyperplasia.^21^, ^22^, ^23^ Meanwhile, mild preoperative symptoms and preoperative medication with anticholinesterase have only been reported to be identified as independent predictors for CSR.^21^ In our study, the five‐year cumulative CSR rate was 44.7%, which was similar or even better when compared with former studies. Multivariate Cox analysis identified older age as a negative factor for achieving CSR. Some articles have reported that thymoma patients without MG might develop MG after thymectomy, even after extended thymectomy.^24^, ^25^, ^26^ However, we did not observe postoperative MG development in our study.

Several limitations should be pointed out in this study. First, this was a retrospective study, and the characteristics of patients enrolled may be different from other centers. Therefore, the patient‐selection bias was unavoidable. Second, patients with thymic tumors always have a long‐term survival. A long follow‐up period is essential for sufficiently evaluating the efficacy of surgery for thymic tumors. However, the follow‐up period was relatively short in our study.

In conclusion, MG had little influence on OS and tumor recurrence of thymic tumors. The new TNM stage system was an independent prognostic factor. Incomplete resection and younger age were risk factors for tumor recurrence. Older age was a negative factor of achieving CSR for thymoma patients with MG after extended thymectomy.

## Disclosure

No authors report any conflicts of interest.
